# Reperceiving depression: how trait mindfulness enhances perceived support through improved doctor–patient relationships and stigma alleviation in depressed young adults

**DOI:** 10.3389/fpsyg.2025.1589931

**Published:** 2025-10-14

**Authors:** Danhong Zhu, Yufeng Yang, Jing Wen, Chao Liu

**Affiliations:** School of Journalism and Communication, Huaqiao University, Xiamen, China

**Keywords:** depression, trait mindfulness, doctor–patient relationship, stigma, rumination, perceived social support, reperceiving model of mindfulness

## Abstract

**Objective:**

Depression, a prevalent mental health disorder among global youth, adversely impacts educational attainment, social functioning, and psychological wellbeing. Given the established protective function of perceived social support against depressive symptoms, this study investigates how trait mindfulness enhances such support through three mediating factors: therapeutic alliance perceptions, ruminative responses, and stigma internalization in clinically diagnosed adolescents.

**Methods:**

Guided by the Reperceiving Model of Mindfulness, this study examines the pathways connecting trait mindfulness, rumination, stigma, doctor–patient relationship perceptions, and perceived social support in adolescents with depression. Utilizing online convenience sampling, 569 participants (aged 14–30) meeting clinical depression criteria were recruited. Analytical procedures involved: Assessing measurement reliability and demographic variations using SPSS 26.0. Implementing structural equation modeling with Amos 26.0 to evaluate model fit, examine latent variable associations, and estimate standardized path coefficients.

**Results:**

The analysis demonstrated that trait mindfulness significantly enhanced perceived social support (β = 0.331, *p* < 0.001), with perceptions of the doctor–patient relationship partially mediating this relationship (indirect effect = 0.023, 95% CI [0.001–0.057]). Trait mindfulness also markedly reduced stigma (β = −0.375, *p* < 0.001), which subsequently diminished perceived social support (β = −0.177, *p* < 0.01). Stigma further mediated the mindfulness-social support linkage (indirect effect = 0.051, 95% CI [0.018–0.097]). In contrast, rumination showed no significant direct effect (β = −0.083, p =0.206) nor mediation capacity between trait mindfulness and social support (indirect effect = 0.040, 95% CI [−0.027 to 0.110]).

**Conclusion:**

This investigation establishes that trait mindfulness effectively augments perceived social support in depressed adolescents through dual pathways: enhancing doctor–patient relationship perceptions and mitigating stigma. Notably, rumination demonstrates no significant impact on social support acquisition in this clinical population. By delineating these mechanistic pathways, our findings highlight mindfulness-based interventions' therapeutic potential, proposing targeted training protocols to amplify social support networks for improved mental health outcomes in youth depression management.

## 1 Introduction

Depressive disorders have become a global public health priority among adolescents, with China experiencing particularly acute challenges as an emerging economy (Han et al., 2021). Projections indicate depressive conditions will constitute the primary disease burden in China by 2030 (Miller and Kirschbaum, 2019), underscored by national epidemiological data revealing 8.3%−16.2% prevalence of psychological disorders (Xiaoli et al., 2014), and approximately 30% exhibiting subclinical depressive symptoms among minors (Li et al., 2010). Official statistics from China's National Health Commission estimate 30 million under-17 adolescents affected by affective disorders, predominantly major depressive disorder (Xiaoli et al., 2014). This mental health crisis manifests through multidimensional impairments (Huang et al., 2021), including academic deficits, neurocognitive dysfunction, social maladjustment, and heightened suicide risks (Li et al., 2022), consequences exacerbated by progressively earlier depression onset. The escalating prevalence, currently affecting 16.2% of secondary students based on meta-analytic findings (Tang et al., 2019). The prevalence of depression intervention among young people in China remains low, only 9.5% of people with depressive disorders in China were treated, and only 0.5% were adequately treated, national programs are needed to remove barriers to availability, accessibility, and acceptability of care for depression in China (Lu et al., 2021). Necessitates urgent development of evidence-based youth interventions targeting symptom mitigation and functional recovery.

Perceived social support networks constitute critical protective factors for depressed individuals, particularly through familial and peer reinforcement mechanisms (Caplan, 1974). Youth social support systems typically comprise four functional domains: familial, peer-based, occupational, and pedagogical support (Rosenfeld et al., 2000). Empirical evidence confirms these networks' therapeutic effects through self-esteem enhancement and stress buffering (Shumaker and Hill, 1991), with familial reinforcement particularly fostering therapeutic optimism through heightened self-worth (Thomas et al., 2017). The buffering hypothesis (Alloway and Bebbington, 1987) posits that perceived social support operates through two synergistic mechanisms: cognitive reappraisal processes that minimize perceived stressor severity, coupled with strengthened self-efficacy beliefs in coping capacities. This dual-pathway model finds empirical support in recent mediation analyses demonstrating how adaptive problem-solving strategies reduce threat appraisal magnitudes (Acoba, 2024). Ultimately attenuating stressor impacts through psychocognitive restructuring. While neurophysiological research confirms support systems' biomodulatory effects on stress response systemsel (Uchino, 2006).

These multilayered mechanisms-spanning psychological, behavioral, and physiological domains—substantiate perceived social support's preventive and interventional value in youth depression management. Enhancing perceived social support utilization among depressed youth can be effectively facilitated through mindfulness cultivation. Rooted in Eastern meditation practices (Marlatt and Kristeller, 1999). Mindfulness involves purposeful, non-judgmental attention to present-moment experiences (Kabat-Zinn, 1994), widely implemented across occupational and educational contexts, demonstrate multifaceted benefits including enhanced work engagement, improved professional attitudes, strengthened organizational trust, and boosted performance outcomes based on current empirical evidence (Johnson et al., 2020). Empirical evidence from positive psychology research demonstrates its therapeutic value through anxiety and depression reduction in young populations (Gong et al., 2023), achieved by fostering metacognitive awareness that promotes non-reactive symptom observation (Chadwick et al., 2009). Although pharmacotherapy often surpasses psychotherapy in symptom reduction, mindfulness-based approaches uniquely enhance interpersonal functioning (Klerman et al., 1974). This psychological construct emerges as a robust predictor of psychosocial wellbeing, showing significant correlations with improved emotional adjustment (Kiafar et al., 2019), and strengthened social support perceptions (Klainin-Yobas et al., 2016). Through cultivating present-centered awareness, mindfulness enables adaptive reappraisal of social interactions, thereby serving as an intrinsic mechanism influencing depressed individuals' support network engagement capacities.

Although previous research has demonstrated the beneficial effects of mindfulness training in alleviating anxiety and depression while promoting emotional regulation, its potential mechanisms for enhancing the perception and utilization of social support remain unclear, particularly in targeted studies involving depressed youth. The existing literature primarily focuses on how mindfulness improves individual psychological states, yet few studies explore its pathways from the perspective of interpersonal interactions and social support mobilization. Moreover, while most intervention studies prioritize symptom reduction, relatively insufficient attention has been paid to functional recovery, especially the enhancement of social functioning. Therefore, this study aims to address this gap by examining whether and how mindfulness acts as an internal psychological mechanism to improve perceived social support among young adults with depression, thereby offering theoretical and practical guidance for developing more efficient and targeted psychosocial intervention strategies.

## 2 Literature review and hypotheses development

### 2.1 Reperceiving model of mindfulness

The reperceiving model of mindfulness, proposed by Shapiro in 2006, identifies three core mechanisms underlying mindfulness: intention, attention, and attitude. Among these, reperceiving functions as the central mechanism, defined as the capacity to disengage from automatic cognitive-emotional patterns, thereby enabling objective observation of internal experiences and external environments with enhanced clarity (Shapiro et al., 2006). The re-perception model proposes that by training individuals to view psychological phenomena as transient, they can develop greater tolerance for unpleasant internal states, thereby fostering improved emotional regulation and wellbeing. Depression often involves persistent, automatic engagement with negative thoughts through rumination. Within mindfulness practice, re-perception encourages patients to observe these thoughts and emotions intentionally and non-judgmentally, which reduces habitual rumination. This process also enhances the capacity to attend to present-moment cognitive and affective responses, sharpening sensitivity to subtle changes in experience. As a result, individuals achieve clearer awareness of mental and emotional content and develop greater cognitive-emotional-behavioral flexibility. When facing the complex distress associated with depression, patients frequently turn to maladaptive coping strategies, such as suppression, judgment, or avoidance. Re-perception involves viewing conscious experience without emotional distortion, allowing for a more neutral and objective perspective on one's thoughts. It helps individuals cultivate a stable mindful stance from which to process negative emotions, promoting non-reactivity and acknowledgment of their temporary nature. Empirical studies have established that mindfulness-based interventions effectively reduce depressive symptoms, including anxiety, relapse risk, acute depressive episodes, and stress. Additionally, these interventions are associated with improved health outcomes, decreased psychological distress, and enhanced overall quality of life (Hofmann and Gómez, 2017). For individuals with depression, this mechanism facilitates detachment from maladaptive emotional states, allowing them to reframe illness-related experiences neutrally, which reduces negative disease cognitions while improving perceived social support. Through this reperceiving process, mindfulness may counteract disease-induced cognitive distortions and fortify social support networks. Empirical applications of this model span multiple domains: mental health outcomes (Brown et al., 2015), emotion regulation processes (Garland et al., 2009), occupational burnout interventions (Liu et al., 2021), and interpersonal relationship dynamics (Dong et al., 2020). Nevertheless, critical gaps persist in understanding how mindfulness specifically enhances perceived social support among depressed young adults through reperceiving mechanisms. Furthermore, as the model primarily derives from mindfulness intervention studies, the applicability of trait mindfulness within this theoretical framework remains underexplored. To address these limitations, this study applies structural equation modeling (SEM) grounded in the reperceiving model to examine mindfulness's dual capacity to alleviate negative disease-related cognitions and strengthen perceived social support in clinical populations.

#### 2.1.1 Mindfulness and social support

Previous research indicates that mindfulness enhances emotion regulation capacities (Liu et al., 2020), enabling more effective emotional management (Siegel et al., 2009). This improvement subsequently facilitates interpersonal relationship development and strengthens perceived social support networks. Individuals with heightened mindfulness exhibit greater environmental attunement, allowing comprehensive integration of internal and external experiences. Such awareness enhances the utilization of social support resources, thereby improving mental health outcomes (Gordon et al., 2020). The mindfulness-perceived social support relationship may be moderated by post-traumatic stress levels. Under disease recurrence and treatment adherence pressures, mindfulness exerts stronger influences on perceived social support among depressed individuals (Kuhl and Boyraz, 2017). Cultural analyses reveal that Chinese populations influenced by mind-body unity traditions frequently manifest psychological distress through somatic symptoms rather than psychological complaints (Chan et al., 2015). Within China's collectivist cultural framework, individuals with severe mental illnesses demonstrate increased self-blame and reduced self-acceptance (Chien and Thompson, 2014). Paradoxically, the cultural emphasis on mind-body integration may render Chinese populations receptive to mindfulness meditation interventions (Liu et al., 2022). Current mindfulness research in China primarily focuses on three cohorts: university students (Zuo et al., 2023), clinical populations (Jing et al., 2021), and family systems (Wang et al., 2021). Notably, mental illness applications remain underexplored, with meta-analyses identifying only 23 relevant studies (Tao et al., 2021). This reveals critical research gaps regarding mindfulness interventions for Chinese youth with depression. Existing trait mindfulness frameworks for depressed young adults predominantly derive from Western cultural paradigms, lacking localization adaptations. Consequently, trait mindfulness effects on perceived social support may yield culturally divergent outcomes between Chinese and Western populations.

Based on the above literature review, we propose the following hypothesis:

H1: Trait mindfulness has a positive effect on perceived social support in young depressed people.

#### 2.1.2 Mindfulness, doctor–patient relationship, and social support

The doctor–patient relationship constitutes a unique interpersonal dynamic established between healthcare providers and patients during medical treatment, founded upon core principles of trust, communication, empathy, respect, and collaboration to enhance patient health outcomes (Emanuel and Emanuel, 1992). This relational framework not only improves patient satisfaction and institutional trust but also strengthens treatment adherence through seven empirically validated pathways: ensuring care accessibility, expanding health literacy (Chen et al., 2023), reinforcing support networks, enhancing emotional regulation, activating social resources, optimizing clinical decision-making (Huang et al., 2023), and promoting patient autonomy (Street et al., 2009). Although initially conceptualized in oncology contexts (National Cancer Institute, 2007), these mechanisms demonstrate particular therapeutic relevance for depression management, where positive clinical interactions can foster psychological wellbeing and adaptive cognitive reframing.

Healthcare providers' mindfulness levels significantly influence relationship- building efficacy. Clinicians demonstrating elevated trait mindfulness tend to adopt patient-centered communication styles, establishing emotionally supportive therapeutic environments conducive to care quality improvement (Beach et al., 2013). Mindfulness practice cultivates dual benefits by generating positive affective states while reducing occupational stress (You and Liu, 2022), thereby enabling healthcare professionals to sustain empathic engagement critical for optimal patient interactions (Amutio-Kareaga et al., 2017). Empirical evidence further identifies clinician self-compassion as a foundational competency that enhances patient-directed compassion through mindfulness training (Epstein and Privitera, 2016).

Current research predominantly examines physician-centric dimensions of medical relationships, analyzing clinical decision-making patterns and empathy development from practitioners' perspectives. This asymmetrical focus overlooks patients' active role as emotional agents with distinct therapeutic expectations and feedback mechanisms (Huang et al., 2022; Zhang et al., 2024). To address this critical gap, our investigation specifically evaluates how depression-afflicted adolescents' mindfulness capacities modulate their perceptions of clinical relationships and subsequent social support internalization.

Based on the above literature review, we propose the following hypothesis:

H2: Trait mindfulness has a positive effect on the perception of the doctor–patient relationship among young adults with depression.
H3: Perception of the doctor–patient relationship has a positive effect on the perceived social support among young adults with depression.
H4: Perception of the doctor–patient relationship mediates the relationship between trait mindfulness and perceived social support.

#### 2.1.3 Mindfulness, rumination, and social support

Nolen-Hoeksema conceptualizes rumination as a cognitive-behavioral pattern wherein individuals persistently focus on depressive symptomatology and its etiology, a process generating maladaptive responses through sustained negative affect (e.g., hopelessness) and behavioral indecision (Nolen-Hoeksema and Morrow, 1991). This cognitive style persists beyond depressive episodes, compromising social functioning by fostering self-isolation tendencies and diminished social support perception (Pearson et al., 2010). Rumination-induced interpersonal dysfunction manifests through three interconnected mechanisms. Chronic emotional overflow from sustained negative immersion frequently escalates interpersonal conflicts, while pessimistic cognitive schemas characterized by despair and apathy tend to evoke social rejection. Concurrently, the cumulative burden of affective-cognitive overload often precipitates active social disengagement, creating cyclical patterns of relational deterioration (Nolen-Hoeksema et al., 2008). Analogous to stigma, ruminative processing erodes support networks through dual pathways: impairing objective resource availability while distorting subjective support appraisal, even when adequate resources exist.

Emerging evidence suggests mindfulness-based interventions constitute an effective therapeutic approach for mitigating rumination-related distress (Chiou et al., 2022). By cultivating present-moment awareness through mindfulness skill development, individuals acquire cognitive flexibility to disengage from perseverative thought patterns (Gu et al., 2022). Mindfulness-Based Stress Reduction (MBSR) demonstrates particular efficacy in this regard, supplanting maladaptive cognitive processes with metacognitive awareness mechanisms that disrupt depressive recurrence cycles (Segal et al., 2012). This therapeutic modality not only reduces rumination frequency but also attenuates associated aggression through cognitive decoupling mechanisms (Borders et al., 2010). Crucially, MBSR alleviates depressive symptoms by reducing rumination, a causal pathway empirically validated through longitudinal clinical trials (van Aalderen et al., 2012). The transdiagnostic applicability of mindfulness training extends to mood disorder populations, where meditative practice decreases rumination propensity while enhancing emotional regulation capacity. These findings collectively indicate that mindfulness interventions effectively modify depression-maintaining cognitive factors through systematic attentional retraining.

Based on the above literature review, we propose the following hypothesis:

H5: Trait mindfulness has a negative impact on rumination in young adults with depression.
H6: Rumination has a negative impact on perceived social support in young adults with depression.
H7: Rumination mediates the relationship between mindfulness and perceived social support in young adults with depression.

#### 2.1.4 Mindfulness, stigma, and social support

Individuals with mental disorders constitute one of the most stigmatized populations globally (Stuart, 2008), particularly within collectivist cultural contexts like China where stigma intensity exceeds Western countries (Xu et al., 2017). This social phenomenon creates substantial barriers to treatment adherence and functional recovery (Gaebel and Baumann, 2003) manifesting through treatment avoidance behaviors and post-recovery social exclusion that heightens relapse risks. The stigmatization process undermines social support systems through interconnected pathways of psychological disengagement and relational attrition. Shameinternalization initiates self-isolation patterns (Di et al., 2021), while perceived inadequacy suppresses help-seeking behaviors. Concurrently, sustained caregiver strain gradually exhausts available support reserves, creating synergistic erosion of interpersonal resources (Mickelson, 2001). Additionally, the prolonged burden on support network members and the depletion of support resources can lead to a gradual decline in perceived social support over time (Eckenrode and Wethington, 1990). This cyclical dynamic generates reciprocal reinforcement between stigma and support network deterioration, wherein diminished social support exacerbates psychological distress, thereby intensifying stigma perception.

Current research predominantly examines perceived social support's buffering effects against stigma, while underinvestigating stigma's corrosive impacts on support networks. Psychological flexibility is defined as an individual's capacity to adapt to changing circumstances, accept distressing thoughts and emotions without avoidance, and act in accordance with personal values (Hayes et al., 1999). A primary goal Acceptance and Commitment Therapy (ACT) is to enhance psychological flexibility, thereby enabling individuals to accept rather than struggle with challenging thoughts and emotions, ultimately facilitating a meaningful life (Koparal and Kiraz Avci, 2025). Mindfulness emerges as a critical cognitive modulator in this context, demonstrating capacity to disrupt maladaptive information processing patterns through enhanced present-moment awareness (Yang et al., 2023). By cultivating non-judgmental acceptance of mental health conditions, mindfulness-based interventions foster metacognitive detachment from societal prejudices, enabling stigma resistance through dual pathways of self-compassion cultivation (Chan et al., 2018), and psychological flexibility enhancement (Yang and Mak, 2017), these mechanisms not only reduce stigma internalization but also improve support network utilization by modifying cognitive appraisals of relational stressors. The resultant cognitive transformation enhances perceived social support sustainability, making mindfulness training particularly efficacious for breaking stigma-support deterioration cycles in depressive populations.

Based on the above literature review, we propose the following hypothesis:

H8: Mindfulness can mitigate stigma in young adults with depression.
H9: Stigma has a negative impact on perceived social support in young adults with depression.
H10: Stigma mediates the relationship between mindfulness and perceived social support in young adults with depression.

**Figure 1:**
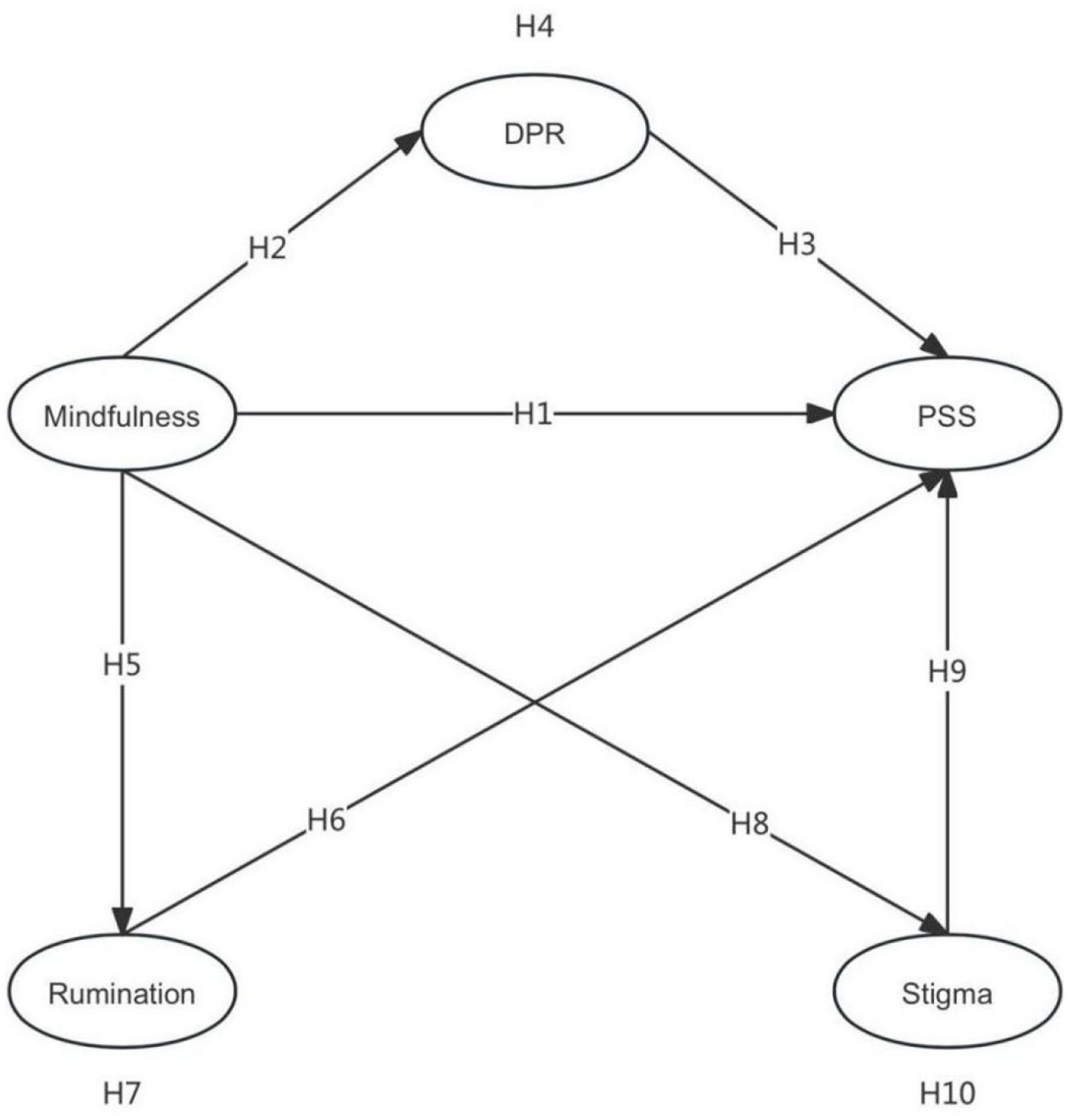
Hypothesis Model. DPR, Doctor–patient relationship; PSS, Perceived social support.

In summary, the hypothesized model of this study is as follows (Figure 1).

## 3 Method

### 3.1 Participants

Methodological rigor was ensured through systematic verification processes. Data collection was restricted to administrator-certified online support groups where membership required submission of medical documentation including diagnostic reports and prescription records. Concurrently, supplementary recruitment via social media platforms implemented equivalent verification procedures, requiring participants to provide matching credential materials. These dual validation mechanisms operated synergistically to guarantee participant eligibility while maintaining protocol consistency across sampling channels.

Adolescence represents a critical psychosocial transition period marked by identity exploration and deferred assumption of adult responsibilities (Arnett, 2000). Individuals aged 14–17 construct initial identities through educational decisions and peer engagement (Erikson, 1968), while those aged 25–30 navigate persistent challenges in career establishment, residential stability, and family formation within contemporary society. This extended transition period renders youth particularly vulnerable to psychological crises, justifying our operational definition of youth as encompassing the 14–30 age range.

### 3.2 Data collection

This study examined mindfulness's influence on perceived social support among depressed youth by administering an online questionnaire through Wenjuanxing (www.wjx.cn), China's predominant survey platform, distributed via convenience sampling across depression support groups on popular social media platforms such as QQ, Weibo, Douban, and Xiaohongshu. Participants received 1.5 yuan compensation upon verifying unique IP addresses, adequate response durations, and eligibility criteria compliance. The two-phase data collection protocol incorporated privacy protection measures throughout.

Following the preliminary survey conducted on September 28, 2024, which collected 126 questionnaires through Wenjuanxing (a Chinese online survey platform) from depression support communities yielding 98 valid responses used for instrument refinement based on reliability and validity analyses, the formal phase commenced on October 28, 2024. This involved systematically screening social media groups using keywords like “depression mutual-aid groups” and “online depression support communities,” excluding those established for less than 1 month or having fewer than 50 members. After obtaining administrator approvals from eligible groups, data collection proceeded until February 1, 2025, accumulating 650 submissions. Rigorous data cleaning excluded responses with insufficient completion time, duplicate IP addresses, repeated device submissions, or non-compliance with study criteria, resulting in 569 valid questionnaires and an 87.6% validity rate.

The demographic characteristics of the 569 valid participants are presented in Table 1. Male participants comprised 30.4% of the sample, with females constituting 69.6%, reflecting a 50% higher depression prevalence among females consistent with epidemiological patterns reported in China's Depression Blue Book. Age distribution spanned three brackets: 19–25 years (59.1%), 14–18 years (25.1%), and 26–30 years (15.8%). Residential distribution indicated 78% urban vs. 22% rural residency. Educational attainment showed a bimodal pattern, with middle school (20.4%) and high school (32.9%) graduates collectively representing 53.3% of the cohort, while 41.5% held college/undergraduate degrees and 5.3% possessed postgraduate qualifications. These demographic parameters align with the target population characteristics essential for this investigation.

**Table 1:** Demographic characteristics of participants (*n* = 569).

**Characteristics**	**Demographic information**	**Frequency**	**%**
Antidepressant medication treatment	Yes	481	84.5
	No	88	15.5
Gender	Male	173	30.4
	Female	396	69.6
Place of residence	Urban area	444	78
	Rural area	125	22
Age	14–18	143	25.1
	19–25	336	59.1
	26–30	90	15.8
Education	Junior High School and below	116	20.4
	Senior High School	187	32.9
	Junior college	99	17.4
	Bachelor's degree	137	24.1
	Master's degree	28	4.9
	Doctoral degree and above	2	0.4

The questionnaire employed in this study comprised six domains: demographic information, trait mindfulness, stigma, perceived social support, rumination, and doctor–patient relationship cognition. All independent variables were measured using five-point Likert scales with differential anchoring: trait mindfulness employed reverse scoring (1 = Very often to 5 = Never), whereas rumination utilized standard frequency scaling (1 = Never to 5 = Very often). Stigma, perceived social support, and doctor–patient relationship assessments shared identical agreement-based anchors (1 = Strongly disagree to 5 = Strongly agree). To ensure measurement validity, all scales were adapted from psychometrically validated instruments documented in established literature. Given the Chinese cultural context, a rigorous back-translation procedure was implemented to achieve linguistic appropriateness and cultural relevance of the translated scales.

### 3.3 Measures

#### 3.3.1 Mindfulness

Trait mindfulness was assessed using the Mindful Attention Awareness Scale (MAAS) developed by Brown and Ryan (2003), a unidimensional scale widely used to evaluate individual levels of mindfulness. The MAAS focuses on attention and awareness in daily life (Baer et al., 2006). It is applicable to both meditators and non-meditators and has good discriminative power. The MAAS conceptualizes mindfulness as an individual tendency, where trait mindfulness is a general quality that individuals possess (Park et al., 2013), the MAAS contains 15 items evaluating cognitive, emotional, and physiological aspects of daily awareness. Previous studies have shown that the scale has good reliability and validity among Chinese adolescents (Black et al., 2012). For enhanced ecological validity with youth populations, four contextually appropriate items were selected through pilot testing. The description of certain measurement items is as follows: I break or spill things because of carelessness, not paying attention, or thinking of something else. The adapted subscale demonstrated robust reliability (Cronbach's α = 0.826, *M* = 2.0615, SD = 0.99601).

#### 3.3.2 Doctor–patient relationship

Doctor–patient relationship cognition was assessed using the Antidepressant Compliance Questionnaire (ADCQ) developed by Demyttenaere et al. (2004). The 33-item scale comprises four dimensions: doctor–patient relationship cognition, autonomy maintenance, positive beliefs, and family support. For this study focusing on depression patients, four items from the doctor–patient relationship cognition subscale were systematically selected. The description of certain measurement items is as follows: My doctor has made me feel confident that antidepressants are the suitable treatment for my depression. The adapted subscale demonstrated robust reliability (Cronbach's α = 0.870, *M* = 3.3196, SD = 1.00803).

#### 3.3.3 Perceived social support

Perceived social support was measured using Zimet et al.'s Scale of Perceived Social Support (MSPSS) (Zimet et al., 1988) comprising three source-specific subscales: family, friends, and significant others. The original 12-item instrument (4 items per subscale) was adapted through pilot testing, with five contextually relevant items selected for this depression-focused investigation. Higher composite scores reflect greater perceived support levels. The description of certain measurement items is as follows: There is a special person with whom I can share my joys and sorrows. The refined scale exhibited acceptable reliability (Cronbach's α = 0.775, *M* = 2.6863, SD = 1.02323).

#### 3.3.4 Stigma

Stigma levels were assessed using the Internalized Stigma of Mental Illness Scale (ISMI) developed by Ritsher et al. (2003) This instrument evaluates subjective internalized stigma experiences among individuals with mental disorders and their family members, with particular emphasis on self-stigma manifestations. The original scale comprises five dimensions: alienation, stereotype endorsement, perceived discrimination, stigma resistance, and social withdrawal. Following established psychometric evidence indicating the stigma resistance subscale's comparatively weaker reliability, this dimension was excluded from the current investigation. Four representative items were systematically selected (one from each retained dimension). The description of certain measurement items is as follows: Nobody would be interested in getting close to me because I have a mental illness. The refined scale exhibited acceptable reliability (Cronbach's α = 0.825, *M* = 3.3774, SD = 1.02361).

#### 3.3.5 Rumination

This study used the Ruminative Responses Scale (RRS) developed by Nolen-Hoeksema (1991), which measures depressive individuals' cognitive focus on self-referential symptoms, causal attributions, and consequential thinking. The original instrument comprises three dimensions: symptom rumination, reflection, and brooding. Through systematic item refinement during pilot testing, the adapted scale demonstrated strong psychometric performance in formal administration. The description of certain measurement items is as follows: Think about how passive and unmotivated you feel. The refined scale exhibited acceptable reliability (Cronbach's α = =0.869, *M* = 4.0492, SD = 1.00192).

### 3.4 Data analysis methods

The collected data were analyzed using SPSS 26.0 and AMOS 26.0 statistical packages. SPSS facilitated descriptive statistics, univariate ANOVA, independent *t-*tests, and reliability assessments, while AMOS enabled confirmatory factor analysis and structural equation modeling to examine variable relationships through path coefficients (β), determination coefficients (*R*^2^), and effect size metrics (*f*^2^). Model fit indices were concurrently analyzed to validate the theoretical framework's empirical adequacy.

## 4 Data analysis results

### 4.1 Differential test of demographic characteristics

To examine demographic differences in mindfulness and perceived social support among young adults with depression, this study conducted one-way ANOVA and independent samples *t-*tests using SPSS 26.0. Independent samples *t-*tests were performed for antidepressant medication use, gender, and residence, while one-way ANOVA was applied to age and education level, following homogeneity of variance testing. A chi-square test *p* < 0.05 indicated non-homogeneous variances, whereas *p* > 0.05 validated ANOVA results (Brown and Forsythe, 1974). As shown in Table 2, no significant differences emerged in perceived social support regarding antidepressant treatment, gender, or residence. Age demonstrated a significant association with perceived social support (*p* < 0.05, *R* = 0.17), explaining 2.9% of the variance, indicating weak predictive power. Education level showed stronger significance (*p* < 0.001, *R* = 0.270), accounting for 7.3% variance and moderate influence on perceived social support.

**Table 2:** Differential testing of demographic characteristics in perceived social support (*n* = 569).

**Characteristics**	**Demographic information**	**Mean**	**SD**	***T* or *F***	**Cohen's *d* or *R***	***P***
Antidepressant medication treatment	Yes	2.684	0.982	−0.123	1.024	0.902
	No	2.701	1.230
Gender	Male	2.707	1.088	−0.398	1.024	0.691
	Female	2.746	1.055			
Place of residence	Urban area	2.743	1.045	0.357	1.024	0.721
	rural area	2.704	1.136			
Age^*^	14–18	2.532	1.027	4.061	0.170	0.018
	19–25	2.774	1.075			
	26–30	2.907	1.045			
Education^***^	Junior High School and below	2.371	1.051	7.131	0.270	^***^
	Senior High School	2.738	1.020			
	Junior college	2.609	1.087			
	Bachelor's degree	2.990	1.024			
	Master's degree	3.310	0.947			
	Doctoral degree and above	4.000	1.414			

^*^p < 0.5, ^***^p < 0.001.

### 4.2 Exploratory factor analysis and confirmatory factor analysis

The references in this study were selectively adjusted according to the research scope while ensuring adequate citation of existing studies and established scales. These scales were validated through exploratory factor analysis (EFA) and confirmatory factor analysis (CFA). Exploratory factor analysis (EFA) was conducted on all scale items using SPSS 26.0. The Kaiser–Meyer–Olkin (KMO) measure of sampling adequacy was 0.857 (>0.7), and Bartlett's test of sphericity yielded a *p*-value of 0.000 (< 0.01), confirming the suitability for factor analysis. Using principal component analysis with varimax rotation, five factors with eigenvalues exceeding 1 were identified after six iterations, collectively explaining 71.7% of the total variance, surpassing the 60% threshold. Procedural controls for common method bias were implemented through anonymous responses and reverse-scored items. The collected data were examined using Harman's single-factor test, with unrotated exploratory factor analysis extracting five factors exhibiting eigenvalues greater than 1. The largest factor accounted for 30.34% of the variance (below the 40% threshold), indicating no substantial common method bias in this study.

Confirmatory factor analysis (CFA) was performed using AMOS 26.0. All factor loadings ranged from 0.616 to 0.886 (see Table 3), exceeding the 0.5 threshold, indicating strong correlations and convergent validity for each latent variable measurement. These results confirm that the questionnaire comprises well-constructed items with appropriate psychometric properties.

**Table 3:** Factor loadings.

**Variables**	**Items**	**Factor Loadings**
MAAS	MAAS1	0.739
	MAAS2	0.664
	MAAS3	0.726
	MAAS4	0.830
PSS	PSS1	0.600
	PSS2	0.815
	PSS3	0.755
Rumination	RRS1	0.857
	RRS2	0.844
	RRS3	0.790
Stigma	ISMI1	0.679
	ISMI2	0.811
	ISMI3	0.752
	ISMI4	0.839
DPR	DPR1	0.676
	DPR2	0.802
	DPR3	0.886
	DPR4	0.797

MAAS, mindfulness; DPR, Doctor–patient relationship; PSS, Perceived social support.

### 4.3 Reliability and validity testing

Internal consistency reliability was assessed using Cronbach's alpha coefficient, with values ranging from 0.775 to 0.870 across variables, indicating satisfactory reliability and internal consistency. Convergent validity was evaluated through factor loadings, composite reliability (CR), and average variance extracted (AVE). All factor loadings exceeded 0.5, CR values surpassed 0.7, and AVE estimates were above 0.5, confirming adequate convergent validity. Discriminant validity was verified by comparing the square roots of AVE values with inter-factor correlation coefficients. The observed correlations between latent variables remained lower than corresponding AVE square roots, demonstrating appropriate discriminant validity (see Table 4).

**Table 4:** Results of validity and reliability.

**Variable**	**Mindfulness**	**DPR**	**Stigma**	**Rumination**	**PSS**	**AVE**	**CR**	**Cronbach's *a***
Mindfulness	**0.742**					0.551	0.830	0.826
DPR	0.098	**0.794**				0.630	0.871	0.870
Stigma	−0.375	−0.037	**0.773**			0.597	0.855	0.852
Rumination	−0.632	−0.062	0.237	**0.831**		0.690	0.869	0.869
PSS	0.480	0.353	−0.332	−0.353	**0.729**	0.531	0.770	0.755

The bold numbers on the diagonal represent the square root of AVE.

AVE, Average Variance Extracted; CR, Construct reliability; DPR, Doctor–patient relationship; PSS, Perceived social support.

The above analysis confirmed the reliability and validity of the measurement tools used in this study. To further evaluate the adequacy of the model design, structural equation modeling (SEM) was conducted for additional analysis.

### 4.4 Model fitting

The structural equation model (SEM) was constructed using AMOS 26.0 and evaluated against established fit criteria: absolute fit indices (GFI > 0.8, SRMR < 0.08, AGFI > 0.8), incremental fit indices (NFI > 0.8, IFI > 0.8, CFI > 0.8), and parsimony indices (*C*_min_/d*f* : 1–5, PGFI > 0.5). As shown in Table 5, the model demonstrated excellent fit with *C*_min_/d*f* = 2.942, SRMR = 0.053, RMSEA = 0.058, PGFI = 0.682, GFI = 0.933, AGFI = 0.909, CFI = 0.951, NFI = 0.928, IFI = 0.951, and TLI = 0.940, meeting all recommended thresholds and confirming its appropriateness for the sample data.

**Table 5:** Model fitting.

**Fit metrics**	***C*_min_/d*f***	**SRMR**	**RMSEA**	**PGFI**	**GFI**	**AGFI**	**CFI**	**NFI**	**IFI**	**TLI**
Value	2.942	0.053	0.058	0.682	0.933	0.909	0.951	0.928	0.951	0.940

### 4.5 Hypothesis testing and mediation effect analysis

**Figure 2:**
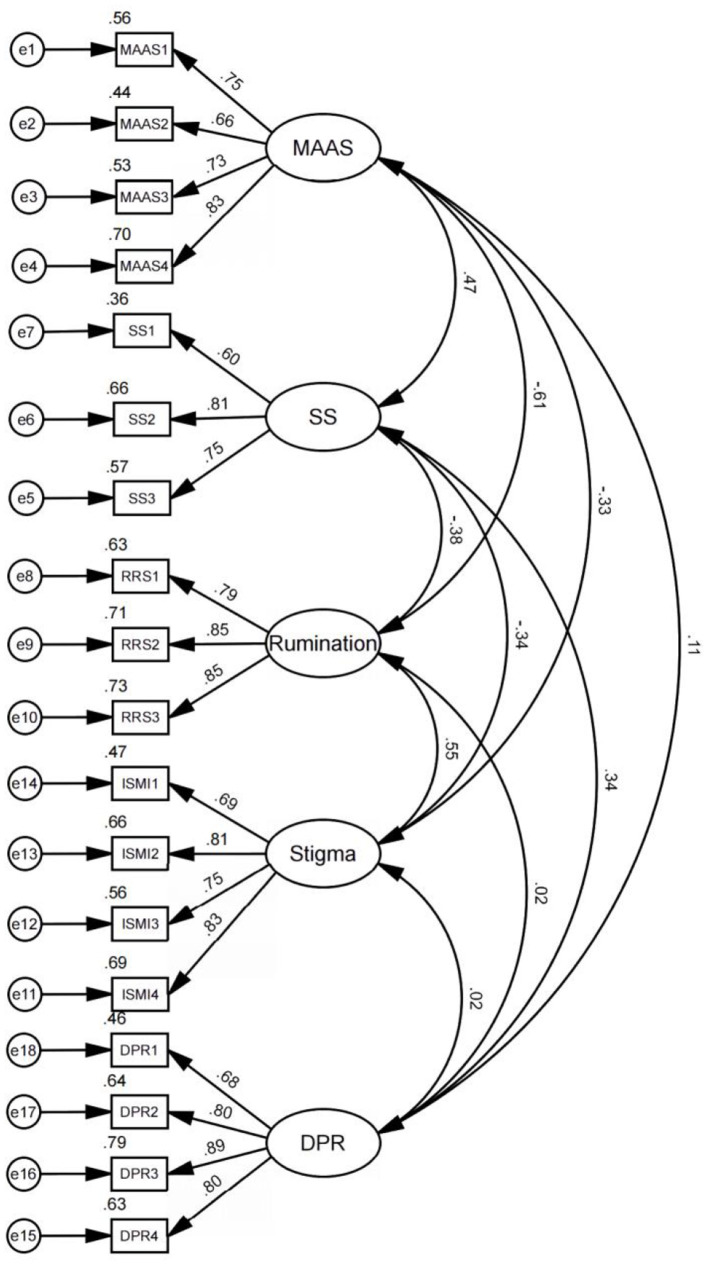
Confirmatory factor analysis. MAAS, Mindfulness; DPR, Doctor–patient relationship; PSS, social support.

**Figure 3:**
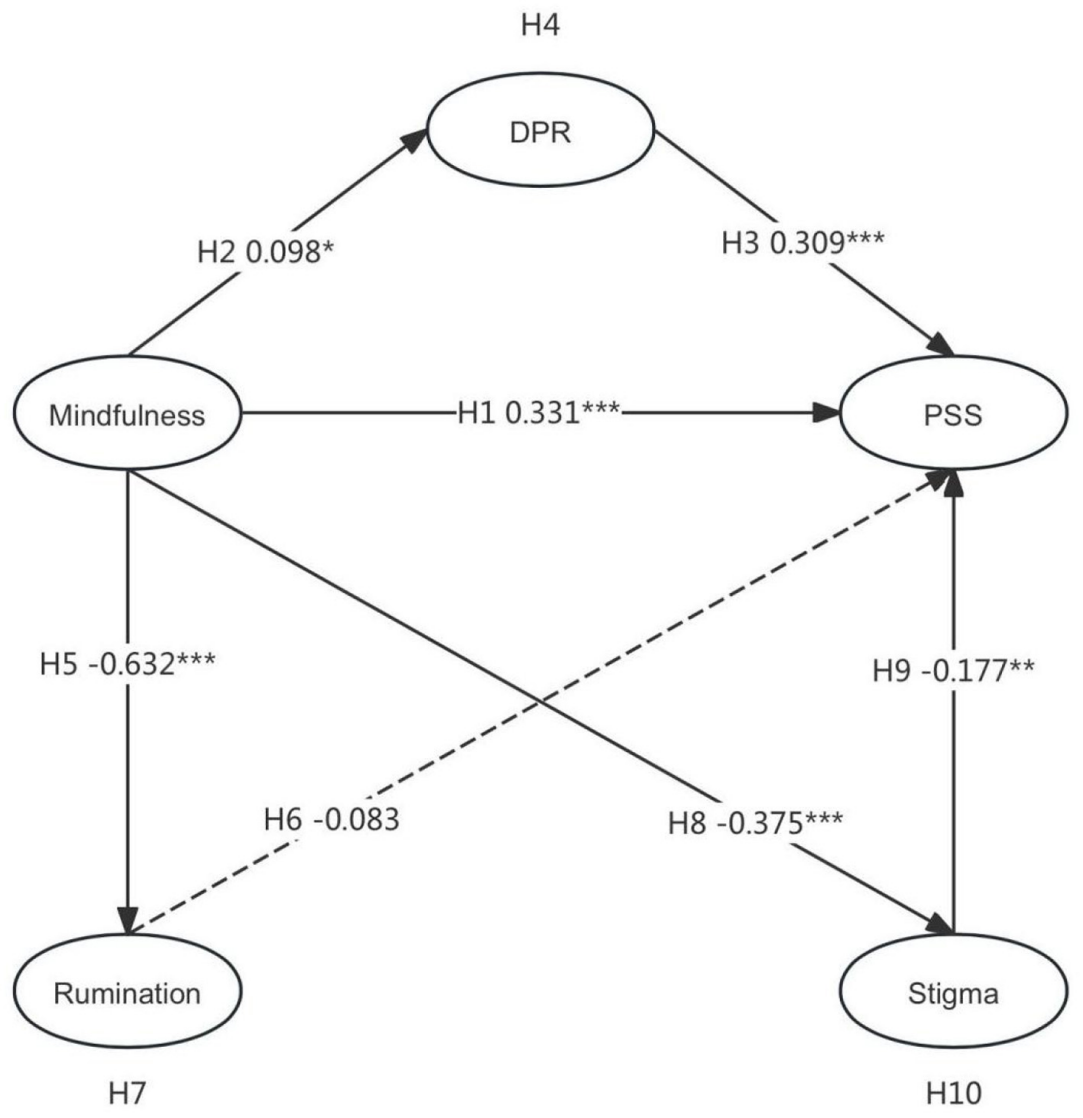
Path coefficients of the proposed model. DPR, Doctor–patient relationship; PSS, perceived social support. *P < 0.5, **P < 0.01, ***P < 0.001.

The hypothesized pathways were analyzed through structural equation modeling (SEM) in AMOS 26.0, with results demonstrating significant relationships for six paths (*p* < 0.05). Trait mindfulness exhibited a positive effect on perceived social support (β = 0.331, *p* < 0.001), supporting H1, while also enhancing doctor–patient relationship cognition (β = 0.098, *p* < 0.05), thereby confirming H2. Doctor–patient relationship cognition further strengthened perceived social support (β = 0.309, *p* < 0.001), validating H3. Trait mindfulness reduced stigma (β = −0.375, *p* < 0.001), consistent with H8, and stigma inversely impacted perceived social support (β = −0.177, *p* < 0.01), aligning with H9. Additionally, trait mindfulness diminished rumination (β = −0.632, *p* < 0.001), substantiating H5. However, rumination showed no significant effect on perceived social support (β = −0.083, p = 0.206), rejecting H6. These validated and refuted pathways are summarized in Figure 2. These validated and refuted pathways are summarized in Figure 3 and Table 6.

**Table 6:** Hypothesis testing results.

**Path**			**β**	**Unstd**.	**S.E**.	**C.R**.	**P**	**Hypothesis test results**
Mindfulness	—>	DPR	0.098	0.079	0.039	2.043	0.041	Support H2
Mindfulness	—>	Rumination	−0.632	−0.570	0.046	−12.416	^***^	Support H5
Mindfulness	—>	Stigma	−0.375	−0.288	0.040	−7.265	^***^	Support H8
DPR	—>	PSS	0.309	0.291	0.050	5.820	^***^	Support H3
Mindfulness	—>	PSS	0.331	0.252	0.053	4.746	^***^	Support H1
Rumination	—>	PSS	−0.083	−0.070	0.055	−1.264	0.206	Not support H6
Stigma	—>	PSS	−0.177	−0.176	0.054	−3.256	0.001	Support H9

^***^P < 0.001. DPR, Doctor–patient relationship; PSS, Perceived social support.

Mediation effects were examined using the Bootstrap method with 5,000 resamples to assess the indirect pathways linking trait mindfulness to perceived social support through doctor–patient relationship cognition, stigma, and rumination (Table 7). The Mindfulness → Doctor–patient relationship Cognition → Perceived Social Support pathway demonstrated a significant indirect effect (β = 0.023, 95% CI [0.001, 0.057]), with the exclusion of 0 from the confidence interval confirming mediation, thereby supporting H4. Conversely, the Mindfulness → Rumination → Perceived Social Support path showed a non-significant indirect effect (β = 0.040, 95% CI [−0.027, 0.110]), as the interval encompassing 0 negated mediation, rejecting H7. The Mindfulness → Stigma → Perceived Social Support pathway revealed significant mediation (β = 0.051, 95% CI [0.018, 0.097]), with 0 excluded from the confidence interval, validating H10.

**Table 7:** Mediation effect test.

**Path**	**Effect type**	**Effect Size**	**SE**	**BootstrapCI (95%)**		**Hypothesis test results**
				**Lower**	**Upper**	
Mindfulness —>DPR —>PSS	Direct effect value	0.252^***^	0.063	0.141	0.390	Support H4
	Indirect effect value	0.023^*^	0.014	0.001	0.057	
	Total effect value	0.275^***^	0.067	0.157	0.421	
Mindfulness—>Rumination—>PSS	Direct effect value	0.252^***^	0.063	0.141	0.390	Not Support H7
	Indirect effect value	0.040	0.020	−0.027	0.110	
	Total effect value	0.292^***^	0.068	0.193	0.441	
Mindfulness—>Stigma—>PSS	Direct effect value	0.252^**^	0.063	0.141	0.390	Support H10
	Indirect effect value	0.051^***^	0.034	0.018	0.097	
	Total effect value	0.303^***^	0.055	0.187	0.456	

^*^P < 0.5, ^**^P < 0.01, ^***^P < 0.001DPR, Doctor–patient relationship; PSS, Perceived social support.

## 5 Discussion

### 5.1 Mindfulness is a predictor of perceived social support (H1)

The study results supporting Hypothesis H1 demonstrate that trait mindfulness positively influences perceived social support among depressed young adults. As articulated in the reperceiving model (Shapiro et al., 2006). This meta-cognitive capacity enables individuals to disengage from entrenched cognitive-emotional patterns through shifting from content-based to process-oriented consciousness. Such cognitive restructuring initiates three fundamental mechanisms—enhanced self-regulation, clarified value systems, and improved cognitive-emotional-behavioral flexibility which collectively improve emotional regulation capacities and promote psychological wellbeing. For depressed youth, this reperceiving ability facilitates clearer recognition of existing support systems, simultaneously increasing network satisfaction and reducing negative reciprocity. Particularly within China's cultural context, mental illness stigma exhibits spillover effects where patients' stigma contaminates family members through vicarious stigmatization (Liao et al., 2019), potentially destabilizing kinship-based strong-tie networks. However, empirical evidence confirms mindfulness's dual function in cultivating interpersonal relationships (Pratscher et al., 2018) and mitigating social anxiety (Hu et al., 2024). Chinese youth support systems comprise both strong-tie (familial) and weak-tie (peer-based) networks (Varga and Zaff, 2018). When strong-tie networks become compromised, mindfulness-enhanced social skills enable compensatory expansion of weak-tie networks, particularly for obtaining emotional sustenance. This contrasts sharply with low-mindfulness individuals who exhibit reduced sensitivity to social support availability and utilization efficiency (Malpass et al., 2012). Therefore, elevated trait mindfulness levels in depressed young adults directly correlate with enhanced perceived social support.

### 5.2 The mediating effect of doctor-patient relationship (H2, H3, H4)

The results of the study, consistent with Hypotheses H2, H3, and H4, reveal that trait mindfulness exerts a positive influence on doctor–patient relationship cognition, which in turn enhances perceived social support, with this cognitive construct mediating mindfulness' effect on support perception. Existing literature documents that mindfulness cultivation among healthcare practitioners reduces burnout (Malik and Annabi, 2022), improves psychological wellbeing (Scheepers et al., 2020), and elevates care quality (Braun et al., 2019), all critical components for fostering therapeutic alliances. Complementing these provider-focused studies, our patient-centered perspective demonstrates that depressed young adults' trait mindfulness similarly improves their physician interaction perceptions, thereby addressing research perspective imbalances. Unlike treatments for other medical conditions, psychological interventions characteristically integrate pharmacological and psychotherapeutic approaches. Adolescent patients undergoing psychotherapy also demonstrate heightened perception of social support from healthcare providers compared to those receiving treatment for non-psychiatric conditions. During this crucial developmental stage marked by heightened emotional needs (Stanton-Salazar and Spina, 2005), depressed youth exhibiting trust in clinicians tend to engage more proactively in therapeutic interactions, increasing receptivity to professional emotional support. This population particularly benefits from skill-based psychotherapeutic approaches (Langer et al., 2021), a preference aligned with Social Cognitive Theory's emphasis on observational learning and self-regulatory mechanisms (Bandura, 1986). Positive physician relationship perceptions create supportive environments facilitating adaptive self-schemas and behavioral patterns—critical precursors for enhanced support network utilization. Notably, constructive doctor-patient cognition enables greater therapeutic engagement, increasing patients' acceptance of clinical recommendations and willingness to seek help from both professional and personal networks. This mechanism operates bidirectionally: while trait mindfulness cultivates positive medical interactions, these improved relationships reciprocally strengthen social support perceptions. Through this mediational pathway, mindfulness ultimately amplifies depressed youths' support network efficacy and psychological wellbeing.

### 5.3 The mediating effect of rumination (H5, H6, H7)

The findings confirmed hypothesis H5, indicating that trait mindfulness negatively predicts rumination. Previous research demonstrates that trait mindfulness alleviates ruminative thinking, particularly maladaptive rumination (Coffey et al., 2010). Specifically, mindfulness helps individuals break habitual rumination cycles by fostering non-judgmental awareness of thoughts and emotions without becoming entangled in them (Nolen-Hoeksema et al., 2008). Contrary to hypotheses H6 and H7, rumination showed nonsignificant associations with perceived social support among depressed youth and demonstrated no mediating effect between trait mindfulness and social support. The direct effect (ES = 0.252) exceeded the indirect effect (ES = 0.04), suggesting rumination may not critically influence social support perception in this population. Trait mindfulness directly enhances perceived social support without requiring rumination reduction. Within China's collectivist context, families remain primary support sources for youth, providing consistent and substantial assistance. Cultural expectations regarding family support may explain why discrepancies between expected and received support rarely trigger rumination in this group (Wei and Huang, 2024). Despite elevated rumination levels (*M* = 4.049, SD = 1.002) among respondents, this may reflect cultural and clinical factors rather than support network deficiencies (Bai et al., 2014). Eastern cultural traditions encourage self-reflection for moral cultivation and self-improvement, potentially elevating reported rumination. Additionally, depressed youth may employ rumination as metacognitive coping strategy (Watkins and Moulds, 2005). Thus, rumination manifests primarily as cultural cognitive patterns rather than diminishing perceived social support within China's distinctive familial support systems.

### 5.4 The mediating effect of stigma (H8, H9, H10)

The study results, consistent with Hypotheses H8, H9, and H10, demonstrating that trait mindfulness negatively predicts stigma levels, which subsequently mediate its effect on perceived social support. Through acceptance and non-judgmental awareness, trait mindfulness enables depressed young adults to resist external stigma while reducing its internalization as self-stigma (Li et al., 2020), thereby alleviating stigma-related distress. Within China's unique cultural context, illness-related stigma exhibits contagion effects, potentially transferring from patients to families as affiliate stigma (Liao et al., 2019). Social face sustains reciprocal relationship networks in Chinese society, and its loss triggers social exclusion and diminished resource access (Yang, 2008). Depressed adolescents may perceive their condition as bringing familial disgrace due to such affiliate stigma, consequently withdrawing from reciprocity networks. Paradoxically, despite actual availability of support resources, stigma-induced self-exclusion reduces receptiveness to assistance, effectively removing themselves from support systems. Previous research indicates that mindfulness enhances cognitive flexibility, thereby facilitating adaptive reappraisal of adversities and positive experiences (Garland et al., 2015). Mindfulness also generates positive affective states while reducing cognitive demands during emotional processing (Chen et al., 2022). For depressed youth, trait mindfulness improves stress reappraisal capacities related to their condition, decreasing negative thought frequency and fostering objective self-evaluations. By promoting stigma reappraisal, trait mindfulness mitigates its detrimental effects on social support acquisition.

## 6 Conclusions

This study investigated young adults with depression to examine how trait mindfulness affects their perceived social support and mediating mechanisms. Results revealed that trait mindfulness enhances perceived social support by improving negative cognitions through cognitive reperceiving. This process enables depressed youth to develop objective understandings of doctor–patient relationships, thereby fostering constructive healthcare interactions that strengthen social support through direct assistance and learned coping strategies. Within China's collectivist context, although vicarious stigma may disrupt support from strong-tie networks, cultivating mindfulness skills helps patients access weak-tie network resources. Additionally, cultural and developmental factors may explain why rumination showed no significant impact on perceived family support among Chinese youth.

This study theoretically validates the applicability of the mindfulness reperceiving model. Although originally developed within mindfulness intervention frameworks requiring meditation training, the findings indicate that individuals with inherent trait mindfulness can employ cognitive reperceiving to mitigate negative cognition and strengthen perceived social support without formal training. The research confirms this model's relevance for depressed young adults by elucidating how mindfulness enhances social support perception through improved doctor–patient relationship cognition and stigma reduction, thereby expanding known pathways of trait mindfulness's social cognitive influence. While prior research predominantly focused on Western populations, this investigation addresses the scarcity of culturally contextualized studies by examining trait mindfulness-social support dynamics among Chinese youth with depression. Furthermore, it rectifies the research perspective imbalance in patient-provider relationship studies, demonstrating that patients' trait mindfulness facilitates constructive therapeutic alliances which subsequently enhance perceived social support.

Societally, this research reveals trait mindfulness's capacity to stabilize and expand social support networks, which proves essential for constructing robust mental health ecosystems and facilitating patients' social functioning recovery. By emphasizing trait mindfulness's critical role in fostering therapeutic alliances, the findings provide actionable insights for optimizing doctor–patient relationships. Findings establish that improved relationship cognition directly strengthens perceived social support through enhanced therapeutic engagement and resource accessibility. Clinically, future psychotherapeutic approaches should incorporate ethical frameworks for delivering emotional scaffolding while equipping patients with skills to mobilize external support systems. Evidence further indicates pharmacological limitations, highlighting psychotherapy's unique advantages in cognitive restructuring. Comprehensive depression treatment.

## 7 Limitations and prospects

This study has several limitations that require further investigation. The questionnaire distribution through social media platforms may have led participants to focus on platform specific experiences rather than real life contexts in their responses. Additionally, using standardized scales without adapting them to the developmental characteristics of young adults may have reduced ecological validity. Future research should modify scale items to better align with participants actual circumstances and develop assessments tailored to this age group. Additionally, given that the current study's use of convenience sampling and recruitment solely from online mutual aid communities may introduce selection bias, the findings should be generalized to broader populations of individuals with depression with caution. Future studies could adopt more randomized and diverse sampling methods to validate these results. This study has identified several mechanisms potentially shaped by cultural context, including family support dynamics in collectivist societies, stigma manifestations, and doctor-patient interaction patterns. These findings hold particular relevance for settings sharing similar Chinese family structures, social identity models, and medical cultural frameworks. Nevertheless, caution remains necessary when generalizing these conclusions to other cultural environments. Future studies should validate these findings across diverse cultural contexts to differentiate universal mechanisms from culture-specific pathways. While this study applied the mindfulness reperceiving model to establish the connection between trait mindfulness and perceived social support in depression, it did not explore the specific metacognitive mechanisms involved. Given the complexity of mindfulness concepts, quantitative designs are needed to clarify these mechanisms.

The current findings on the positive effects of trait mindfulness in doctor–patient relationship cognition should be expanded to examine underlying mediators. In the future, we can explore the role of positive thinking in offline medical settings and expand the applicability of mindfulness interventions. Furthermore, this study employs a cross-sectional design, which precludes the establishment of causal relationships between variables. It is plausible that supportive doctor–patient relationships and lower levels of illness stigma may also enhance patients' trait mindfulness. Although trait mindfulness reflects a relatively stable cognitive and attentional disposition, it can be cultivated through sustained intentional practice. Beyond formal meditation, everyday activities and interpersonal interactions offer opportunities for developing mindfulness. A positive doctor–patient relationship provides a psychologically safe environment in which patients can openly describe their symptoms and emotional experiences. This process encourages patients to turn attention inward, carefully observe bodily sensations, and articulate emotions—practices analogous to “body scanning” and “emotional awareness” in mindfulness training. Thus, a supportive clinical relationship may itself serve as an informal means of cultivating mindfulness. Similarly, reduced stigma disrupts automatic negative thought patterns by creating a cognitive space between experience and reaction. This interruption promotes metacognitive awareness, allowing individuals to observe emotions from a decentered perspective, which further reinforces mindfulness capacities. Considering the ongoing neurocognitive development of young adults, external factors influencing trait mindfulness stability require attention. Tracking changes in mindfulness levels, social support, and mental health outcomes over time through longitudinal studies could clarify causal relationships. Experimental designs may further strengthen causal inferences about mindfulness interventions.

## Data Availability

The original contributions presented in the study are included in the article/supplementary material, further inquiries can be directed to the corresponding author.
